# Using microwave-accelerated digestion instead of dry ashing during sodium analysis of low-moisture, part-skim mozzarella

**DOI:** 10.3168/jdsc.2020-0008

**Published:** 2020-12-11

**Authors:** D.T. Grossbier, Tonya C. Schoenfuss

**Affiliations:** Department of Food Science and Nutrition, University of Minnesota, 1334 Eckles Ave., St. Paul 55108

## Abstract

Mineral analysis of cheese products is a labor- and time-intensive process. Pretreatment using dry or atmospheric wet ashing to decompose organic matter before measurement on a spectrometer can take more than 24 h to complete. Samples of low-moisture, part-skim mozzarella (LMPS) cheese were digested using microwave-accelerated digestion (MAD) and conventional dry ashing. A comparison of 23 samples, in duplicate, was performed to determine equivalency. A two one-sided test (TOST) analysis showed that the confidence intervals were within acceptable differences for standard method variation (less than 70 mg/100 g per International Dairy Federation method 119:2007). Bias was observed for the quantification of sodium in microwave-digested samples, averaging 14 mg/100 g higher. Based on the TOST criteria, we concluded that the methods are equivalent.

## Highlights

• Microwave-accelerated digestion (MAD) is faster than conventional digestion for mineral analysis
• Cheese composition affects dielectric properties of food and can require modification of microwave parameters to achieve complete digestion
• Low-moisture, part-skim mozzarella samples digested by MAD and dry ashing pretreatments were equivalent

## Graphical Abstract

**Figure:**
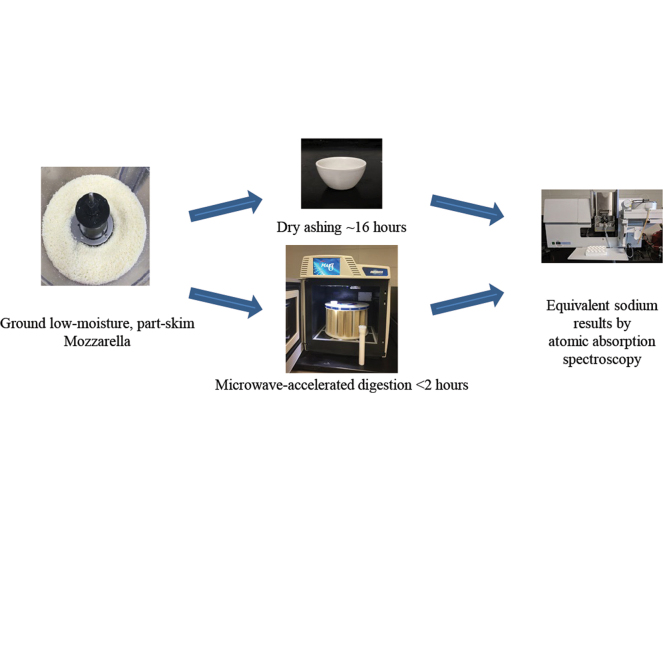
**Summary:** Quantification of minerals in dairy products requires a time-consuming step for decomposition of organic matter. Sodium composition affects functionality, safety, nutrition, and organoleptic properties of cheese. Low-moisture, part-skim mozzarella cheese (LMPS) is the highest volume cheese produced in the United States. We describe a rapid method for the digestion of LMPS using microwave-accelerated digestion (MAD). Results were equivalent between MAD and standard dry ashing for digestion before quantification of sodium by atomic absorption analysis.

Standard methods for mineral analysis such as atomic absorption and inductively coupled plasma-optical emission spectroscopy require a pretreatment to digest organic matter to isolate the inorganic mineral fraction. Traditional methods consist of either dry or wet digestion. Dry digestion uses a controlled combustion procedure referred to as dry ashing (IDF, 2007). The dry ashing pretreatment technique is a time-consuming procedure. Cheese samples are subjected to a 16-h ashing protocol (10 h of ramp time to 550°C followed by a 6-h hold) in addition to cooling, according to the International Organization for Standardization-International Dairy Federation (**ISO-IDF**) method 119:2007 (IDF, 2007). Alternatively, the *Standard Methods for the Examination of Dairy Products* procedure requires a comparably short 6 h in the furnace, but at the cost of a tedious combustion step before ashing (Wehr and Frank, 2004). Both methods require the use of ashing crucibles, which must undergo a 6-h nitric acid bath soak, followed by a drying step, before use.

Atmospheric wet digestion uses a strong acid, most often concentrated nitric acid. This procedure is conducted under atmospheric conditions; however, samples must be covered and boiled for 2 h (Wehr and Frank, 2004). In addition to being time consuming, there are inherent safety risks to both the analyst and laboratory components due to the fumes of the boiling nitric acid.

Pressurized microwave-accelerated digestion (**MAD**) is presented as an alternative preparation method for wet digestion before mineral analysis in ISO-IDF method 119:2007 (IDF, 2007). The details of the procedure in the standard are vague, however, and it is stated that they require tailoring based on the matrix. A study by Schoenfuss et al. (2014) presented a method for MAD of blue cheese. Their reported method was found to be suitable for blue cheese; however, it was not known whether it could be used for other cheeses with different macronutrient compositions and reduced mineral contents without adaptation. The purpose of this study was to compare MAD to dry ashing in the preparation of low-moisture, part-skim mozzarella (**LMPS**) cheese for sodium analysis. We hypothesized that the methods would be equivalent and that modifications of the MAD procedure would be required.

Commercially produced LMPS samples were procured consisting of 4 different brands and production dates, with 23 samples in total, run in duplicate. The outer 1 cm was removed and cubes of 40 mm^3^ were cut using a multi-wire cutter (CubeKing, Redco Food Service Equipment, St. Paul, MN). Samples of 80 ± 2 g were pulsed in a blender equipped with a Mini-Blend jar (Osterizer 6640, Sunbeam Products Inc., Boca Raton, FL) for 10 s followed by 5 s of high-speed blending. Samples were obtained for each method evaluated from this blended sample.

A MARS 6 microwave digestion system (CEM Corp., Matthews, NC) was used for the MAD procedure, which was adapted from the method of Schoenfuss et al. (2014). Representative 0.5-g samples were transferred to a polytetrafluorethylene digestion vessel to which 10 mL of 68 to 70% spectral-grade nitric acid was added. Samples were allowed to stand for at least 15 min before capping and digesting to allow for off-gassing. Samples were digested in a reactor (MARS 6 Microwave Reactor System) using a 20-min linear ramp to 210°C with a 15-min hold time at that temperature. Samples were cooled at room temperature (~30 min), degassed, and opened in a chemical hood before transferring to 50-mL volumetric flasks, which was filled to volume using double distilled water. The total required time to perform the MAD protocol, exclusive of weighing, was approximately 80 min. Power output was automatically adjusted to maintain a linear ramp with an upper limit of 1,300 W. The upper limit power output was increased to 1,300 W from 1,100 W used in the Schoenfuss et al. (2014) method to achieve the desired temperature during ramping. This adjustment was presumably necessary due to differences in the dielectric properties of the cheese because these determine the rate of heating that will occur in a microwave. The blue cheese used in the Schoenfuss et al. (2014) study had a higher sodium concentration range (780 to 1,300 mg of sodium/100 g of cheese), and higher moisture (approximately 49%; Schoenfuss et al., 2014), compared with what would be expected in LMPS (700 mg of sodium/100 g of cheese, 48% moisture; USDA, 2020). Other changes to the microwave parameters that could be considered to achieve a fully digested sample could include decreasing the ramp rate and lengthening the hold time. Alternatively, diluting the samples with water would improve the dielectric properties (increasing the dielectric constant and loss factor), as long as the dilution was accounted for during mineral analysis. We performed serial dilutions using double distilled water to reach an anticipated concentration of 0.5 ± 0.2 ppm sodium (to be within the linear range of the spectrometer). A solution of 27 g/L lanthanum chloride was added to the final dilution to achieve a 10% (vol/vol) concentration. Lanthanum chloride is used to reduce interference during measurement.

Dry ashing was performed according to ISO-IDF method 119:2007 (IDF, 2007). Briefly, 1-g samples were placed in 15-mL acid washed and dried porcelain ashing crucibles. A muffle furnace (Thermolyne F48000, Thermo Fisher Scientific Inc., Waltham, MA) was programmed to achieve a 50°C/h ramp with a 6-h hold at 550°C. After ashing, crucibles were allowed to cool to room temperature, and 1 mL of 69% spectral-grade nitric acid was added. The solution was transferred to 250-mL volumetric flasks. Serial dilutions with double distilled water were performed to reach an anticipated concentration of 0.5 ± 0.2 mg/L sodium. A solution of 27 g/L lanthanum chloride was added to the final dilution to achieve 10% (vol/vol).

Sodium quantifications were performed on a PerkinElmer AAnalyst 100 atomic absorption spectrometer 100 (Perkin-Elmer Inc., Waltham, MA) according to the ISO-IDF method (IDF, 2007). To determine equivalency, results were analyzed using a two one-sided test (TOST) of the mean differences as mentioned by Schoenfuss et al. (2014) and according to Borman et al. (2009).

The sodium concentrations measured by dry ashing analysis ranged from 447 to 908 mg/100 g of LMPS. Sodium concentrations measured using MAD ranged from 441 to 880 mg/100 g. Figure 1 shows the mean values across all samples for dry ashed and MAD treatments.

**Figure 1:**
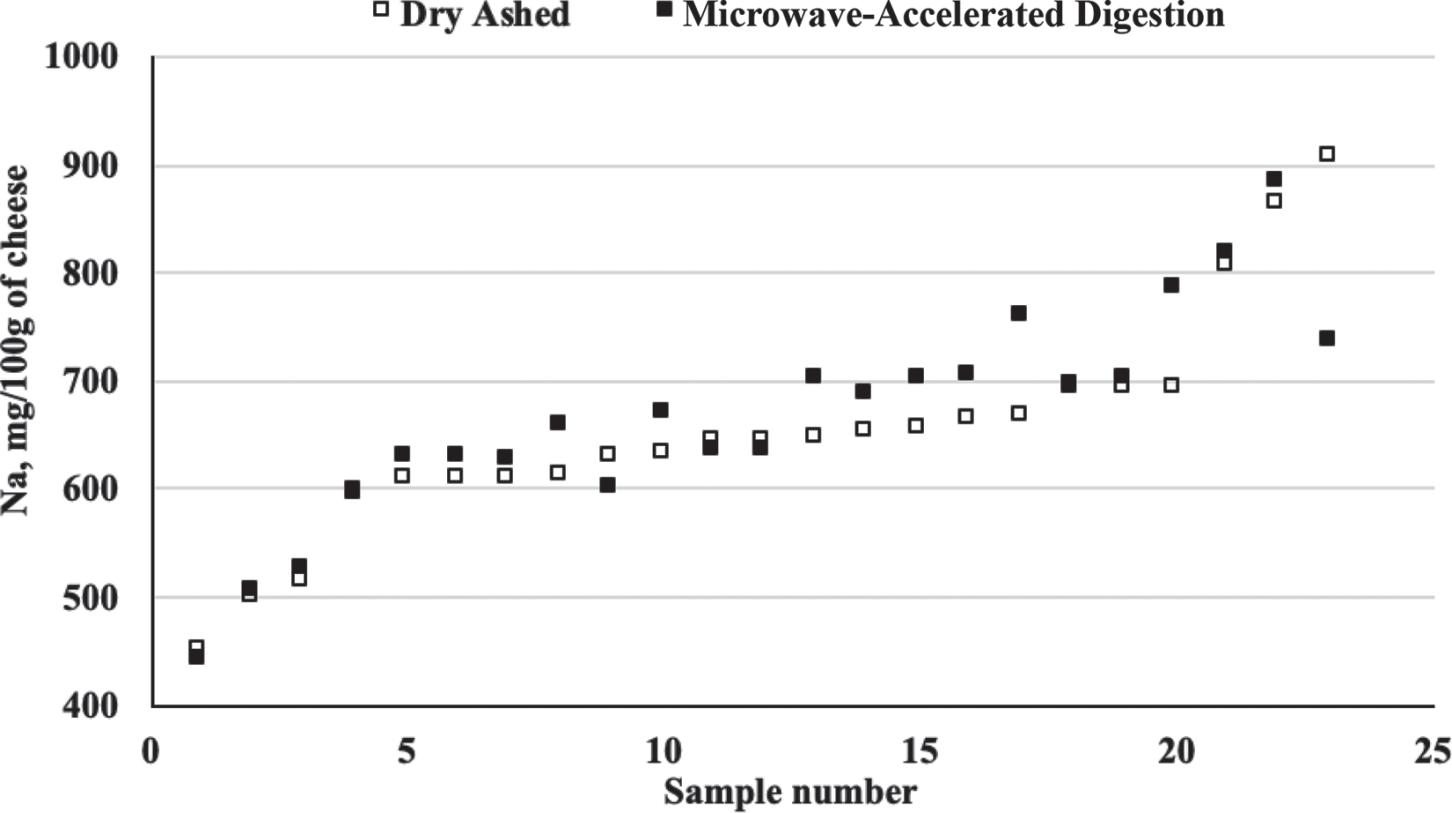
Average sodium content of low-moisture, part-skim mozzarella cheese digested using either dry ashing or microwave-accelerated digestion across all samples (n = 23; average of 2 measurements per method, per sample).

Mean sodium levels for dry ashed and MAD preparation treatments were 653 and 670 mg/100 g, respectively. The broad range of sodium values in commercially available LMPS is similar to that reported by Agarwal et al. (2011). Moistures ranged from 45.2 to 49.9%, with a mean of 47.5%. Total protein was determined to be between 24.7 and 30.3%, with a mean of 28.1%.

Bias was seen with higher observed values for the MAD digestion (Figure 1). The TOST analysis of the mean differences showed no significant difference under the proposed analytics. The ISO-IDF method for sodium in dairy products proposes that the standard deviation of reproducibility (SD_R_) for freeze-dried cheese is 70 mg/100 g (IDF, 2007). As in Schoenfuss et al. (2014), a theta of 11.65% was used, which was determined as 2 times the SD_R._ The 90% upper and lower bounds of the confidence interval of the difference between the means were 14.5 and −50.6, which lies within the ±70 mg/100 g proposed range. With only 3 exceptions, all samples were within this range (mean difference 18.0 mg/100 g). The current study demonstrates that MAD is a suitable substitute for dry ashing in sample pretreatment for sodium analysis of LMPS. We achieved a clear digest with MAD of cheese using nitric acid with a temperature ramp of 50°C/h to 210°C and a 15-min hold. For use with other cheeses with different dielectric properties, such as lower sodium or moisture, microwave parameters such as power output may need to be adjusted to achieve those temperatures. Alternatively, adjusting the dielectric properties of the sample (e.g., diluting with water) or investigating lower temperatures with longer holding times to obtain to achieve complete digestion of the sample could be necessary.
